# MicroRNAome: Potential and Veritable Immunomolecular Therapeutic and Diagnostic Baseline for Lingering Bovine Endometritis

**DOI:** 10.3389/fvets.2020.614054

**Published:** 2020-12-23

**Authors:** Ayodele Olaolu Oladejo, Yajuan Li, Xiaohu Wu, Bereket Habte Imam, Wenxiang Shen, Xue Zhi Ding, Shengyi Wang, Zuoting Yan

**Affiliations:** ^1^Key Laboratory of Veterinary Pharmaceutical Development of Ministry of Agriculture, Lanzhou Institute of Husbandry and Pharmaceutical Sciences of Chinese Academy of Agricultural Science, Lanzhou, China; ^2^Department of Animal Health Technology, Oyo State College of Agriculture and Technology, Igbo-Ora, Nigeria

**Keywords:** endometritis, miRNAs, cytokine, signaling pathways, toll like receptors, gene, miRNA mimics or inhibitors

## Abstract

The bovine endometrium is a natural pathogen invasion barrier of the uterine tissues' endometrial epithelial cells that can resist foreign pathogen invasion by controlling the inflammatory immune response. Some pathogens suppress the innate immune system of the endometrium, leading to prolonged systemic inflammatory response through the blood circulation or cellular degradation resulting in bovine endometritis by bacterial endotoxins. The microRNA (miRNA) typically involves gene expression in multicellular organisms in post-transcription regulation by affecting both the stability and the translation of messenger RNA. Accumulated evidence suggests that miRNAs are important regulators of genes in several cellular processes. They are a class of endogenous non-coding RNAs, which play pivotal roles in the inflammatory response of reproductive diseases. Studies confirmed that miRNAs play a key regulatory role in various inflammatory diseases by mediating the molecular mechanism of inflammatory cytokines *via* signal pathways. It implicates some miRNAs in the occurrence of bovine endometritis, resorting to regulating the activities of some inflammatory cytokines, chemokine, differentially expressed genes, and protein through modulating of specific cellular signal pathways functions. This review dwells on improving the knowledge of the role of miRNAs involvement in inflammatory response as to early diagnosis, control, and prevention of bovine endometritis and consequently enlighten on the molecular improvement of the genes coded by various differentially expressed miRNA through the need to adopt recent genetic technologies and the development of new pharmaceutical preparations.

## Challenges Posed by Postpartum Bovine Endometritis

The world has been battling reduced reproductive performance in dairy cattle caused by uterine disorders. Endometritis is a widely known reproductive disorder in dairy cattle that usually leads to reduced milk production and fertility and a squalled reproductive life wastage (1). Retained fetal membranes is an important phenomenon in the pathogenesis of subclinical endometritis in postpartum dairy cows (2, 3). Recently, Germeyer et al. (4) recorded insufficient endometrial growth marked by reduced endometrial cell proliferation that could lead to reproductive failure. Researchers have documented that bacteria are associated with the uterine disease, using virulence factors that cause tissue damage and cause endometrial inflammation. The ability of various bacteria to inflict persistent inflammatory responses and probable cellular activity changes in bovine endometrium differs based on time of invasion and population of the microbes (5–11). The ability of the organism to fight pathogenic microbes or the durability of the species depends on resistance and tolerance (12–14). The postpartum uterine disease is a polymicrobial disease, and the microbial population in the uterus fluctuates during the postpartum era, with cycles of bacterial infection, elimination, and re-infection (15). The bacteria that most commonly grow in animals with uterine disease are *Escherichia coli, Trueperella pyogenes, Fusobacterium necrophorum, Prevotella*, and *Bacteroides* (16, 17). Gram-positive bacteria have been isolated from the uterine lumen of postpartum cow, which posed a significant influence on the occurrence of inflammation as indicated by Ricci et al. (18). Gram-positive bacteria (*Staphylococcus* species) are the most prevalent pathogen isolated from the uterine lumen in cows and the predominant pathogenic flora in the early to late days of the postpartum period. Triacylated lipoprotein (TAL) or lipothieioc acid (LTA), which were the endotoxins found in the cell walls of Gram-positive bacteria, performs an important role in disease symptom development (19–21). Other Gram-positive bacteria include *Streptococcus* species (18), *Bacillus* spp., and *Corynebacterium pyogenes* (7, 11). The clinical uterine disease appears only when bacterial growth exceeds the competence of the immune system (9, 22). Exceeding the immuno-competence of the uterine tract is mediated by the cellular reaction and associated changes in key inflammatory molecular mediators, genes, and protein postpartum and reaffirms that sustained inflammation is a key feature of endometritis in dairy cow. Recently, there has been an attempt to identify the molecular signatures associated with subclinical or clinical endometritis in cattle (23). The level of inflammatory cytokines and transcriptome profile of changes in bovine endometrium is important in understanding the effect of endometritis on uterine gene expression and changes in dairy cows. Endometrial cell miRNAs control the development of inflammatory cytokines that contribute closely to regulating gene expression by either degrading the mRNA or inhibiting the translation of proteins and are involved in various biological processes and believed to be diagnostic markers for preeclampsia and multiple types of cancer (24–26). The miRNome profile and associated molecular signal pathways are dysregulated, which disturb the homeostasis of the uterine environment and uterine receptivity, resulting in endometritis. Several types of research have given insight into the role of miRNAs in inflammatory cytokine production and differentially expressed genes and proteins by mediating the function of cellular signal pathways (27–33). In this review, we shall enumerate several works done on some individual miRNAs implicated in endometritis molecular characterization and regulation, how to improve the understanding of the role of miRNA involvement in inflammatory response as to early diagnosis, control, and prevention of bovine endometritis, and consequently enlighten on the molecular improvement of the genes coded by various differentially expressed miRNA through the adoption of recent genetic technologies and development of new pharmaceutical preparations.

## Molecular Expression of RNA in Endometrial Cells

RNA is composed of single-stranded nucleic acids of the four nucleotides A, C, G, and U bound by an alternating residue of phosphate and ribose sugar. It is the first medium in translating DNA information into proteins essential for a cell's functioning. Such RNAs also play a direct role in the metabolism of cells (25, 34). It forms RNA, copying into a single-stranded nucleic acid the base sequence of a portion of double-stranded DNA, called the gene, in transcription. Besides that, the messenger RNA (mRNA) molecules' coding region will translate into proteins; other cell RNA elements are involved in various processes including transcriptional and post-transcription regulation of genetic code, thermal and ligand detection, translation control, and RNA turnover (34, 35). They have shown that some RNA molecules embrace complex protein molecules and act as biological catalysts. It bases most RNA analysis in normal and infectious endometrial cells on RNA coding protein (mRNA and its transcript), with a lack of ribosomal RNA (rRNA) information, transfer RNA (tRNA). rRNA is in the cell cytoplasm where ribosomes are found and directs the transformation of mRNA into proteins. tRNA is in the cellular cytoplasm and is involved in the synthesis of proteins (34, 35). Transfer RNA usually transports amino acids to the ribosome corresponding to the three-nucleotide codons of rRNA and joins amino acids, forming polypeptides and proteins (25). The messenger RNA (mRNA) and its transcripts are related to their function in the characterization of molecular endometrial cells. Besides mRNA, tRNA, and rRNA, RNAs are divided into coding (cRNA) and non-coding RNA (ncRNA) (36). There are two types of ncRNAs, housekeeping ncRNAs (tRNA and rRNA) and regulatory ncRNAs, which are further classified according to their size. Long ncRNAs (lncRNAs) have at least 200 nucleotides, while small ncRNAs have <200 nucleotides (37–41). Among the regulatory ncRNAs are microRNAs (miRNAs), small interference RNAs (siRNAs), piwi-associated RNAs (piRNAs), long non-coding RNAs (lncRNAs), (40) circular RNAs (circRNAs), and tRNAs derived from small RNAs (tsRNAs) (42–44), and the most studied molecules are miRNAs, lncRNAs, and circRNAs, with 60% reported that to be regulated by miRNAs (25, 45). The messenger RNA (mRNA) transcribes the genetic code from DNA into a form that can be read and used to make proteins. The messenger RNA (mRNA) reportedly contained genetic information from the nucleus via the cell's cytoplasm. The relative mRNA expression of the inflammatory cytokine gene has also formed the basis for evaluating the effect of endometritis on the adaptive immune system of postpartum dairy cow (46, 47). Cytokine gene analysis indicated, however, that a similar inflammatory process was occurring in cows with persistent endometritis and those developing endometritis between the first and second month postpartum. Previous investigators (48, 49) reported an increased expression of a variety cytokine genes such as IL-1a, IL-1b, IL-6, IL-8, and CSF-1, pro-inflammatory cytokine genes, and a decreased expression of IL-1RA and IL-10, regulatory genes, in uterine cytobrush and endometrial cells from cows diagnosed with endometritis during the early postpartum period (46, 50). Furthermore, cows with spontaneously resolved endometritis as well as cows that remained free of uterine disease during the first 2 months postpartum displayed a significant down-regulation in the expression of both pro-inflammatory and regulatory cytokine genes (51). There was a decrease in TNFα and IL-1 at week 1 in endometritis cows compared to healthy cows. These two main pro-inflammatory cytokines stimulate the expression of IL-8 and adhesion molecules on vascular endothelial cells, leading to neutrophil and monocyte chemoattraction, and activate neutrophils and monocytes, promoting increased phagocytosis and bacterial killing (49). IL-6 is an important pro-inflammatory cytokine which functions in several aspects of inflammation such as induction of fever, increase in vascular permeability, and induction of acute-phase proteins by the liver. The lowered expression of pro-inflammatory cytokine genes in the peri-partum period in cows that developed endometritis could be due to an intrinsic defect in endometrial cell function (52). Therefore, concurrent regulation and molecular study of these cytokine genes of bovine endometrium based on their production time and reactivity in parturient physiological event in dairy cows may form a basis for the development of preventive therapeutic agent.

## Molecular Characteristics of miRNA of the Endometrium in Different Physiological Conditions

**Figure 1:**
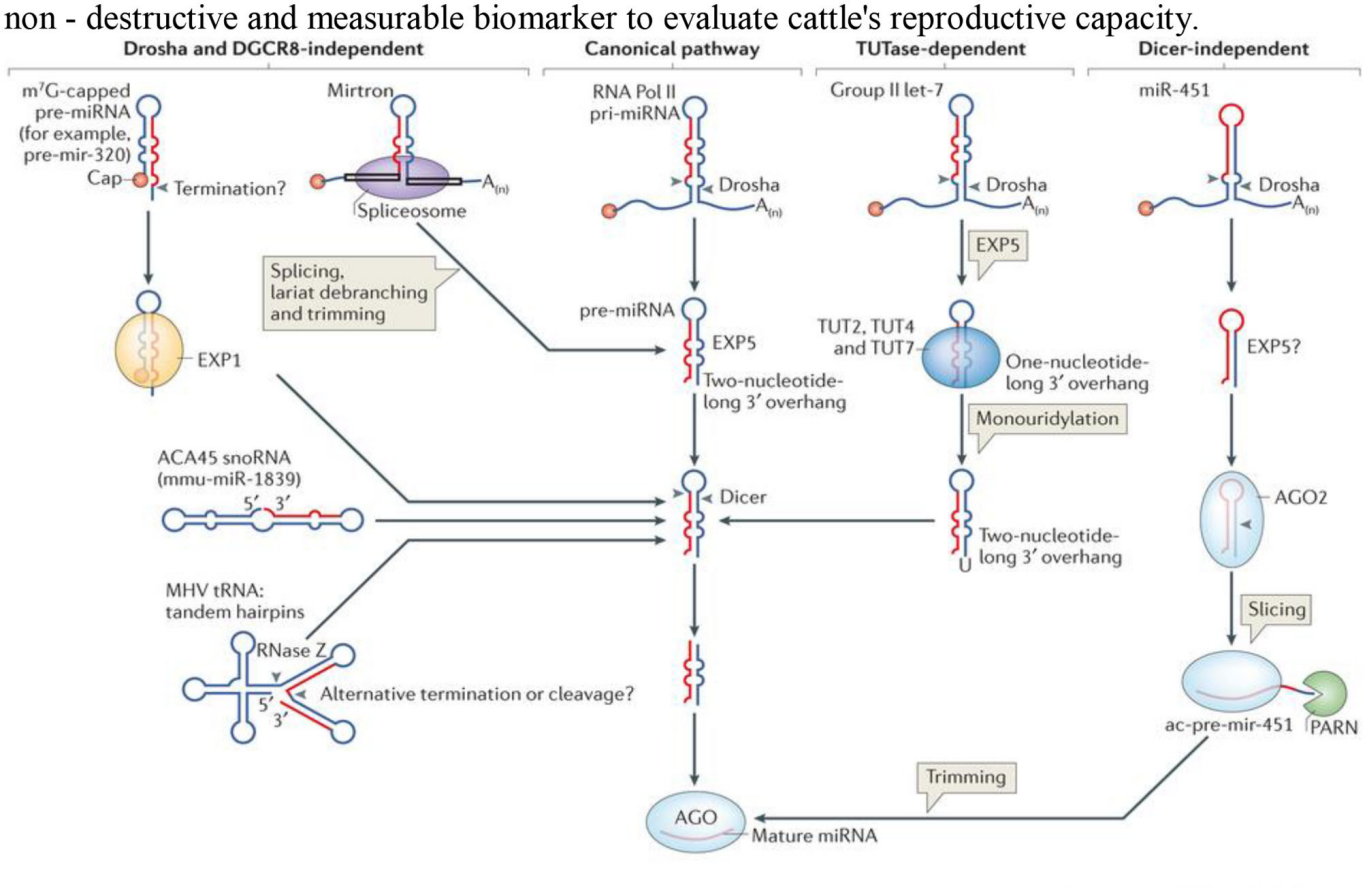
Schematic diagram showing the four different pathways for the biosynthesis of microRNA.

microRNAs (miRNAs) are non-coding RNA with 20–24 nucleotides that function in post-transcription regulation of gene expression in complex organisms, affecting both the stability and the translation of mRNAs (53–55) (Figure 1). The differential coercive capacity of the miRNAs observed in this study may be because of the difference in target gene molecular architecture 30-UTR, which subsequently affects miRNA–mRNA interaction (56). Besides the rule governing miRNA–target mRNA interactions, a conserved seed match composed of miRNA bases 2–9 is a good interaction predictor (57–59), and perfect base-pair matching may not signify a communication between miRNA and the target gene (56) and the G:U base pairs that are at the target sites (57). The number and the arrangement of miRNA recognition sites may also affect the degree and the specificity of the miRNA-mediated gene (55). GO and KEGG pathway review of differentially expressed miRNAs and target genes predicted the likely role of differentially expressed miRNAs in bovine endometrial cell inflammatory response (58). Several studies have stated that miRNAs are important gene regulators in several cellular processes, including inflammation (59, 60). Altered miRNA expression and unnecessary target repression can have various implications, as it may involve these genes in different molecular mechanisms and biochemical properties. Target genes assigned to different functional classes provide crucial input into the uterine functions that are critically impaired by subclinical endometritis, depending on the signal pathways identified (61, 62). Researchers have shown various regulated biological functions where animal miRNAs have a central role in the development of certain diseases and diverse biological properties. Van Rooij and Kauppinen (63) stated that many miRNAs are in pre-clinical and clinical trials as new pharmacological therapeutics for cancer and viral and different inflammatory diseases.

Several researchers have recently documented improvements in the endometrial transcriptome (mRNA) and miRNome profiles of bovine endometritis (54, 64). A recent study showed strong alterations in the endometrial transcriptome (mRNA) and miRNome profiles of cows affected by subclinical or clinical endometritis, which had a major effect on uterine homeostasis and receptivity (27, 65, 66). In the literature review, we find that the specific function of miRNAs in understanding the mechanisms underlying the interactions between innate immune/inflammatory reactions and endometrial physiology and the possible therapeutic function of certain inflammatory agents remain an enigma. Ibrahim et al. (67) argued that lipopolysaccharides (LPS) induced the aberrant expression of miRNAs and their targets, which are involved in the control of uterine homeostasis, leading to endometrial dysfunction. Other researchers (27, 68) have investigated and reported endometrial transcriptome and miRNome changes in the profile and associated molecular pathways caused by subclinical or clinical endometritis. The transcriptome profile alterations were observed in subclinical or clinical endometritis in animals by microarray and qRT-PCR analyses in endometrial cells with subclinical or clinical endometritis equivalent doses of LPS in *vitro*. They found that transcriptomic profiling showed an altered expression level of 203 genes in clinical endometritis (CE) relative to healthy endometrium (HE) species. There are 92 genes, including PTHLH, INHBA, DAPL1, and SERPINA1, which were significantly highly regulated and 111 genes which had significantly poorly regulated expression levels, including MAOB, CXCR4, HSD11B, and BOLA, as recorded in clinical endometritis. The expression patterns of only 28 genes in subclinical endometritis have been substantially altered, of which 26 genes including PTHLH, INHBA, DAPL1, MAOB, CXCR4, and TGIF1 were identified in both subclinical and clinical endometritis. Subclinical endometritis was reported to induce the aberrant expression of miRNAs and deregulation of their respective molecular networks and pathways. This dysregulation of the corresponding target gene networks and canonical and biological pathways suggests the possible regulatory role of uterine miRNAs in the production and progression of bovine subclinical endometritis (27, 62, 67, 68). The expression of miRNAs in endometrial cytobrush samples from healthy cows and cows with subclinical endometritis was performed. A miRNA expression review revealed the dysregulation of 35 miRNAs, including miR-608, miR-526b^*^, and miR-1265 in clinical endometritis animals, and 102 miRNAs, including let-7 families in subclinical endometritis in dairy cows. There are other miRNAs, including let-7e, miR-92b, miR-337-3p, let-7f, and miR-14, found in both HE and CE in the postpartum cow. Further analysis of selected differentially expressed genes and miRNAs in the endometrial stroma and epithelial cells challenged *in vitro* with LPS stimulation showed six candidate miRNAs to be down-regulated both in HE and CE animals (miR-1265, miR-1204, miR-1203, and miR-196b).

Palma-Vera and Einspanier (69) stated that MiR-106a manifestation is regulated by interferon tau and predicted the position of interferon-responsive factor genomic binding sites that can regulate the transcription of genes encoding in the endometrial response to embryonic development, so this explains the essential nature of miRNA in bovine fertility. This study unraveled the alteration of the endometrial transcriptome and miRNome profile in cows affected by subclinical or clinical endometritis. There are 23 overexpression-conveyed miRNAs in subclinical endometritis, potentially target genes that make up a gene network that mediates the production of inflammatory cytokines, notably NF-kB, showing that the regulation of this transcription factor is important in constraining inflammatory responses in postpartum cow uterine lumen. Extended inflammatory response disrupted fundamental endometrial cellular processes influencing uterine receptivity, folliculogenesis, oocyte maturation, and ovulation, eventually contributing to decreased fertility and the need to allocate unknown miRNA transcripts to work. The genomic position of six differentially expressed miRNAs (miR-25, miR-194, miR-423-3p, miR-98, miR-339-5p, and miR-215) overlapped with different transcripts, and several studies have shown that intronic miRNAs are both host-expressed genes (70–73) and produced from a common transcript or that intronic miRNAs and their host genes may support commonly expressed genes (74). Di Pietro et al. (75) also reported an upregulation of miR-27a-3p and miR-124-3p in endometrium and serum during CE and found no association between miR-27a-3p and IGF1 in the endometrium. The receiver operating characteristic curve analysis showed that an endometrial and serum miRNA study could discriminate against women with chronic endometritis. miR-27a-3p and miR-124-3p may be non-invasive CE markers used for the evaluation of endometrial efficiency in *in vitro* fertilization. miR-126, widely expressed in human endothelial cells and vascular tissues, regulates an important step in vasculogenesis (76, 77). miR-423-3p has IKBKB, JUN, INSR, MAPK14, and ID3 mRNA recognition sites in 3-UTR. miR-196 controls four central gene recognition sites in 30-UTR of IKBKB (JUN, INSR, MAPK14, and NOS2 genes), while miR-24-3p has a stronger post-transcriptional effect on bovine subclinical endometritis. The over-expressed miRNAs were highly associated with the Ingenuity Pathway Analysis canonical pathway storage compared to the randomly chosen genes in the list of reference genes (78). MiR-29a, expressed only by follicular cells, was reported to be involved in gene regulation during the early stages of corpus luteum development (79). Similarly, certain miRNAs, such as let-7f and miR-125b, are involved in regulating bovine cyclic reproductive activity (80). Soleilhavoup et al. (81) stated that miR-34c has the potential of a non-destructive and measurable biomarker to evaluate cattle's reproductive capacity.

### miR-223 Role in the Upregulation of Inflammatory Stimulations

NOD-like receptor NLRP3 plays a vital role in a variety of inflammatory diseases, including type 2 diabetes, atherosclerosis, and inflammatory bowel diseases, and the production of immune responses, triggered by a number of endogenous and exogenous agonists; LPS serves as the starting signal for subsequent activation processes and hastens tissue damage (82–85). Increased NF-kB p65 phosphorylation levels have shown an association with increased miR-223 both in endometritis and in bovine endometrial epithelial cells induced by LPS; however, blockage of NF-kB significantly de-regulated miR-223 expression (86). Moreover, miR-223 overexpression suppressed the canonical NF-κB pathway of bovine endometrial epithelial cells as reported by Zhou et al. (87). Further reports have it that miR-223 attenuated the production of pro-inflammatory cytokines, which is induced by the activation of the canonical NF-κB pathway and intracellular miR-223 levels promoted through the activation of the NF-κB pathway, which leads to inhibited NF-κB activity with impaired inflammatory processes at a certain level. As a space–time dependent of NF-kB, Mir-223 expression collectively serves to restrict the level of activation of NLRP3 and to protect inflammatory reactions. This pharmacological stabilization of miR-223 upregulation can be a new therapeutic approach of subclinical endometritis and other inflammatory diseases in cows (86–88).

### miR-488 Mediates the Negative Regulation of the Inflammatory Pathway

miR-488 negatively regulates LPS-stimulated endometritis through inhibition of reactive oxygen species (ROS) production and the AKT/NF-kB signal pathway in bovine endometrium. MiR-488 expression decreased on a dose-dependent basis during endometrial inflammation induced by LPS, which suggests a close relationship with the immune response triggered by LPS (89). The pro-inflammatory cytokines' inhibitory expression was adversely associated with the expression of the miR-488 and showed the suppression of the expression of Rac1, and an inhibitory AKT/NF-kB signal mediated LPS activation of endometritis. It also diminished the accumulation of intracellular ROS to prevent the inflammatory reactions of bovine epithelial endometrial cells (BEECs) mediated by LPS (20, 39, 54, 55, 58, 61). The inflammatory response nature of LPS-stimulated BEECs triggers the intracellular accumulation of ROS and modulation of the signaling pathway AKT/NF-kB by miR-488 directly targeting Rac1's negative phase. The miR-488 control of Rac1/AKT/NF-κB signaling inflammatory pathways in other inflammatory diseases, including endometritis, needs further exploration (90, 91).

### miR-148a as a Suppressor of Inflammatory Indicators

We recognize the significant component of miRNAs in the production of reproductive systems in cattle in previous studies (92), miR-148a, which belongs to the miR-148-152 family, a deeply conservative mammalian miRNA. It was documented to be critical for regulating tumor growth, inflammation, and immunity (93, 94). The analysis of data on miRNA sequencing showed that miR-148a distinguished between healthy cows and endometritis (23, 27). Other researchers found that miR-148a expression alteration in bovine mammary epithelial cells with *E. coli* and miR-148a was specific to *E. coli* infection (95, 96). Indeed miR-148a may inhibit DSS-induced colitis in mice. Oddly, the previous study also found that miR-148a expression was reduced in LPS-stimulated BEECs and further indicated that miR-148a may have a significant position in endometritis pathogenesis. Intensive work needs to be carried out in evaluating the prognostic and diagnostic role in physiopathogenesis of endometritis in dairy cattle (93). In essence, the accepted role of various receptors and ligands will be required for evaluation of research reports based on the expression of microRNA implicated during bovine endometritis to understand the molecular physiology and pathogenesis, leading to the provision of novel prevention, control, and treatment of endometritis by the adoption of microRNA-based immuno-molecular diagnosis and therapy.

### The Let-7 Family miRNAs as Molecular Negative Regulators of Inflammatory Cytokines

The role of the Let-7 family miRNA molecular and cellular signaling pathway in relation to bovine endometrium function because of bacterial inflammation needs further research. In the inflammatory process, however, miR-let-7c plays an important role. Zhao et al. (97) stated the regulatory mechanism underlying the let-7c in the pathogenesis of endometritis. This miRNA's over-expression reduced the LPS-induced uterine inflammation that reduced the exposure of pro-inflammatory cytokines by inhibiting the activation of the NF-kB signal pathways. Following LPS-induced injury, miR-let-7c was involved in tissue repair. Jiang et al. (98) found that let-7c functioned to reduce the release of pro-inflammatory cytokines as a negative regulator of inflammatory reactions after LPS-induced inflammatory responses. Hence, the let-7c family may serve as an anti-inflammatory mimic product in the treatment and control of bovine endometritis (97, 98).

### miR-643 Inhibits Lipopolysaccharide-Induced Endometritis Progression

Significantly, an earlier study showed that 23 miRNAs were expressed abnormally in endometritis in which miR-643 expression decreased most but not miR-215 (61). The miR-643 expression in LPS-treated HEECs decreased, implying that the down-regulation of miR-643 may lead to endometritis progression. NF-κB signal activation may cause the expression and release of inflammatory cytokines, including TNF-5-007, IL-1β, and IL-6, resulting in inflammatory injury (99). Some studies have documented various miR-643 targets, including the X-linked apoptosis protein inhibitor and the zinc finger E-box binding of the homeobox transcription factor 1 (100, 101). Zhao et al. (102) argued that, collectively, by targeting the TRAF6 gene, miR-643-attenuated LPS-induced inflammatory response may show a pathway for endometritis immunotherapy treatment.

### miRNA-185 Regulates the VEGFA Signaling Pathway

Retention of fetal membranes (RFM) of cows is an important reproductive disorder that usually results in endometritis in postpartum cows (33). Vascular endothelial growth factor (VEGF) A, reportedly regulated by miRNA-185, may induce the release of arachidonic acid through the signaling pathway that influences RFM (10, 33). With miRNA-185 incriminated by the activation of the VEGFA signaling pathway, the expression levels of most of the investigated genes (VEGFA, PLC, PRK, RAF, MEK, MAPK, and PLA) are reduced particularly by the abnormal expression of P-p44/42 MAPK, which influenced the release of fetal placenta after calving (103). The overexpression of miRNA-185 inhibited the VEGFA signaling pathway, which plays a role in retaining the fetal membrane during postpartum that may lead to endometritis.

### Uterine Exosome microRNA Features in Endometritis

**Figure 2:**
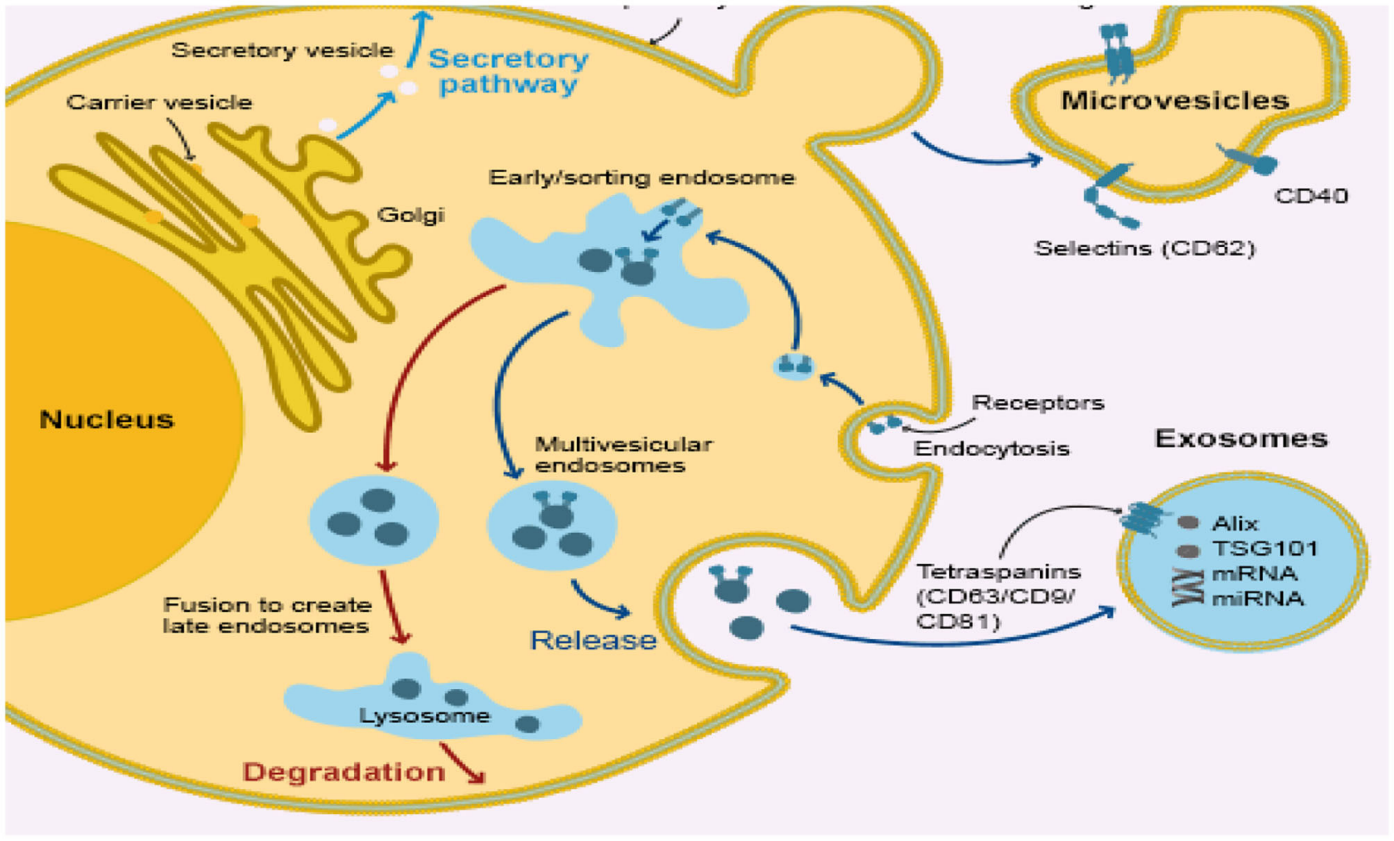
Infected bovine endometrial epithelium cell releasing uterine exosome which contains miRNA.

Exosomes are cup-shaped bilayer phenosomes of the membrane, forming multi-vesicular bodies (MVBs) (104) with a size between 30 and 150 nm via endocytosis (105). Part of those exosomal-containing MVBs are the degrading and lysosome-degraded MVBs and part of those MVBs forms exotic MVBs which merge into the extracellular matrix by the cytoplasmic membrane (Figure 2). These released exosomes (106) are placental, epithelial, dendritic, mast, and T-cells. It can also release exosomes in body fluids, such as serum, urine, amniotic, semen, milk, saliva, and cavity fluid (107). Accumulated evidence suggests the *in vivo* status of many physiological changes or diseases (108–110) in the bio-fluid expression of miRNAs, showing that they can be used as diagnostic markers for humans and infectious diseases, including endometritis (105, 111–113). It may release the exosomes into the uterine cavity *via* the endometrial epithelium to the embryo or adjacent endometrium, which is an important intercellular communication medium, through the transmission of signals, miRNAs and mRNA. Material transfer affects the receptivity, embryonic development, and implantation of endometrial products (114–116). Recent studies have suggested an improvement in the cleavage rate and formation of blastocyst in cloned embryos by adding exosomes isolated from the conditioned medium of somatic cell nuclear transmission embryos (117). The first report also shows that exosomes derived from bovine oviducts can improve the embryo quality (118–123) and enhance the developmental capacity of somatic cells, showing the crucial role that exogenous exosomes play in embryo development (93, 100). The main molecules of exosome miRNA regulation are mainly in receptor cells, which have a gene-silencing role (124, 125). miRNAs represent about 70% of the cellular total miRNAs in exosomes (126, 127). Bovine uterus exosomes affected the development of embryos under endometrial conditions. The identification of this miRNAs was carried out by means of a deep sequence that assessed their pattern of expression in the exosomes from the cavity fluid of healthy cows and endometriotic cows. Three of the most controlled miRNAs and six of the most expressed miRNA applicants were selected as early detection markers for endometritis detection.

## Relationships Between miRNAs and Endometrium Toll-Like Receptors and Antimicrobial Peptides

**Figure 3:**
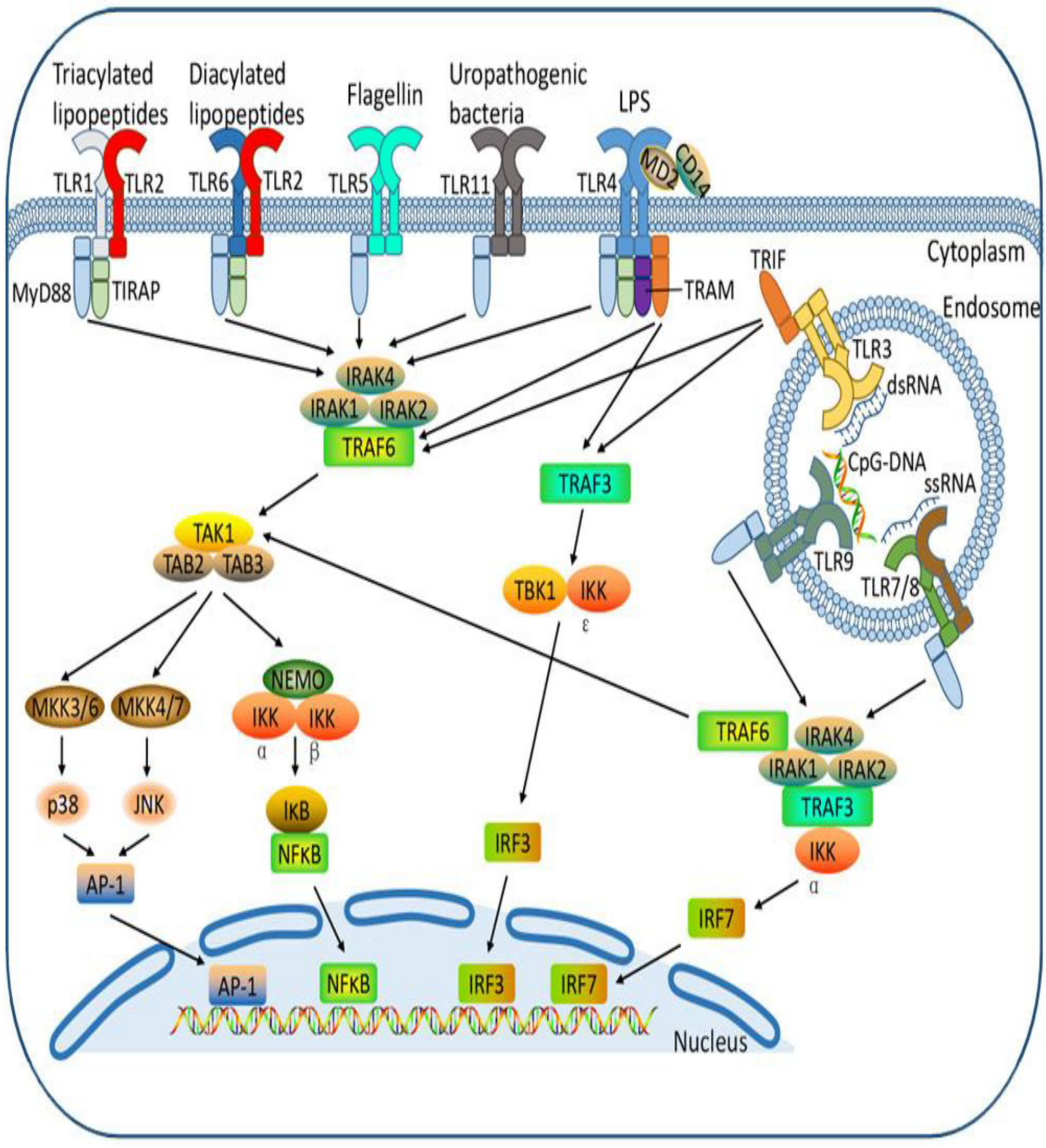
Different Toll-like receptors activating specific cellular signal pathways expressed by different bacteria ligands. The cell receptor activation by bacterial pathogen-associated molecular patterns with cellular interconnectivity of adapters, sensor proteins to influence inflammatory response.

**Figure 4:**
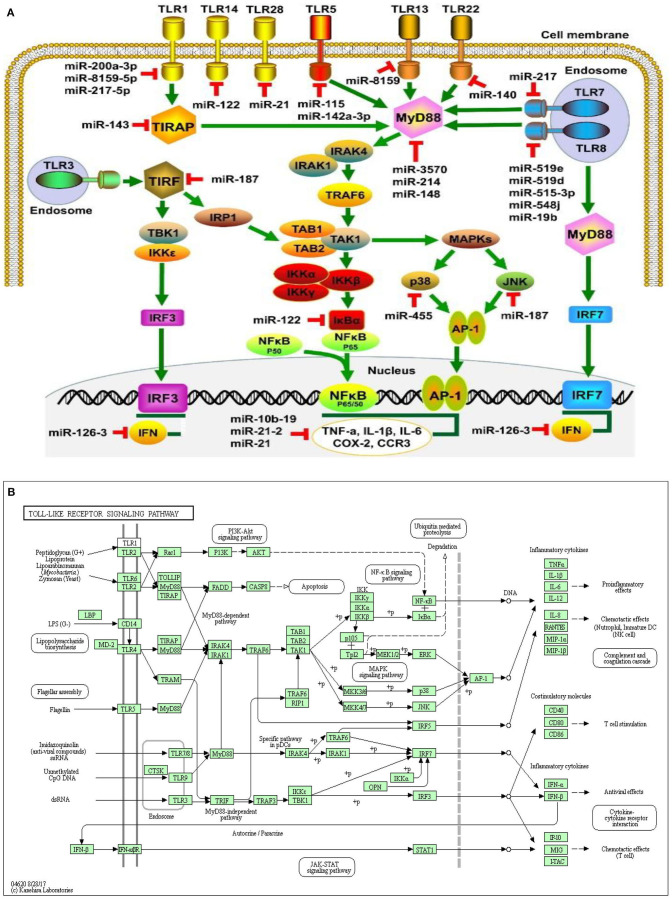
miRNA expression on cellular connectivity and activities with different Toll-like receptor regulation and molecular function of the cellular signaling pathways **(A)**. Toll-like receptor intracellular relationship with various microorganism ligands and activated or functional signal pathway with inflammatory cytokine stimulation **(B)**.

The Toll receptor-like signaling pathway activates key molecules for driving infectious agents to react with immune cells and attracting them to the site of infection (128–130). Antimicrobial peptides (131, 132) are the initial protection against microbes based on innate immune systems, including Toll-like receptors. Pathogen-associated molecular structures are recognized by Toll-like receptors (TLRs), and 10 members of the gene's family are typically expressed (133–135). Bacterial lipids such as LTA stimulate TLR1, TLR2, and TLR6 activation, while nuclear acids, mostly from viruses, adopted TLR3, TLR7, TLR8, and TLR9. TLR4 recognizes gram-negative bacteria such as *E*. *coli* LPS (Figure 3). The bacterial flagellin TLR5 binds and the bacterial DNA TLR9 recognizes (Figure 4A) (134). Cows, mare, ewes, and sows registered gene expressions of TLR1; TLR1 transcripts were detected in the endometrium under earlier reports (136, 137). In clinical endometritis, a substantial up-regulation of TLR 2, indicating its role in infection compared to the estrous process, was observed. The expression of TLR2 in cystic endometrial hyperplasia was documented in canine uterus (138). The TLR2 stimulation by diacylated and triacylated bacterial lipopeptides majorly from Gram-positive bacteria, which stimulate the expression of IL-6 and IL-8 (20), records the development of bovine endometrial cells. Endometrial mRNA upregulation may be induced by the increased expression in endometrial, stromal, and infiltrating leukocytes (139). TLR2 is known to occur throughout endometritis. TLR 2, occurring on the cell surface, attracts bacteria lipopeptide, glycolipid, and peptidoglycan ligand. *T. pyogenes* is a general cause of clinical endometritis in dairy cow (15, 140, 141). During endometritis, TLR6 transcripts increased dramatically and TLR2 constitutes a heterodimer, a link to the ligands to stimulate an inflammatory reaction (142). In bovine endometrial epithelial and stromal cells, TLR6 stimulation by diacylated bacterial lipopeptides upregulates IL-6 and IL-8 (20). Endometritis is regulated by membrane-bound TLRs such as TLR 2, 6, and 10. By contrast, the most commonly down-regulated or non-modulated intracellular TLRs are TLR 3, 7, and 8. The inflammatory endometrium status has been established as being more associated with the membrane-bound upregulation of TLRs (TLR 2, 4, 6, and 10). LTA has no affinity for TLR4 but activates TLR2. TLR4 and TLR2 are both cell surface receptors. TLR4 is unique among the TLRs because it recruits both MyD88/Mal and TRIF/TRAM adaptor proteins; TLR2, on the other hand, recruits just MyD88/Mal pathways. The TLR2/TLR6 complex is activated by LTA and diacylated lipoproteins, but not by triacylated lipoproteins (Figure 4B). Besides cytokines, the principal effector of mucosal defense in pathogens, antimicrobial peptides (AMPs) were found to be involved in the physiological role of the body and had been recognized as part of the innate immune system (143, 144). β-Defensin (DEFB) is a family of AMPs that can permeate bacterial membranes, including DEFB1, DEFB4A, and DEFB5, linguistic AMP, and tracheal AMP (145), which is central to the AMP family. β-Defensins are cationic, small peptides that are formed by three-stranded β-blades on six disulphide-linked cysteines, containing 26–42 amino acids (146). TLR-mediated signaling or the release of inflammatory cytokines (145–147) triggers AMPs in response to infection or injury. Endometrial β-defensin transcripts occur in cows, and the production of AMPs such as TAP and LAP has been stimulated by the treatment of LPS endometrial epithelial cells (139, 147, 148). They have shown endometrial inflammation resolution to be important for endometrial regeneration or regenerative mechanisms by reducing inflammatory and immune-related cell populations, expression of inflammatory mediators and gene expressions, and enzyme proteolytic activity (39, 64, 149).

MicroRNAs were a recently described class of promoters involved in multi-layer enhancement of TLR signaling pathways, including TLR expression modulation, TLR-related adaptor enzymes, signaling molecules, and transcription factors activated by TLR and inflammatory cytokines. Scientific investigations have shown that, in order to control the pathways of TLR signaling and adaptive immune responses, miRNAs play a significant role and act as immunostimulatory agents for diverse cellular processes. Strikingly, a number of miRNAs control several molecules that are active in the TLR signaling pathways which are composed of signaling proteins, regulating molecules, transcription factors, cytokines, and TLRs (150–154). Within the miRNAs, TLR4 expression can be controlled by the let-7 miRNA family, including let-7e and let-7i. The downregulation of TLR4 expression in mouse peritoneal macrophages resulted in increased expression and downregulation of let-7e by miRNA mimics and inhibitors, respectively, leading to overexpression of TLR4 (155). In human biliary epithelial cells, macrophages, and epithelial cells, the Let-7 family controls TLR4 expression (156), possibly due to the variations in the TLR-induced miRNA expression profiles of various cell types. The myeloid-specific miR-223 controlled both TLR4 and TLR3 expression in granulocytes (157). A study showed that TLR4 can also be downregulated by miR-146a, resulting in a macrophage inflammatory response (158). Furthermore, MiR-511 may serve as a presumed positive TLR4 regulator, whereas TLR4 expression appears to suppress monocytes and dendritic cells under similar conditions. (159). In addition, TLR3 targeted expression in rat macrophages and enhanced arthritis by inducing pristane; miR-26a negatively controlled the TLR3 signaling pathway (160). Another receptor regulated by miRNAs is TLR2, whose expression is adversely and positively regulated by miR-146a and miR-105, respectively (154, 161). miR-19a/b upregulates TLR2 expression in fibroblast-like synoviocytes of rheumatoid arthritis patients (86, 162). In addition to decreasing TLR2 protein expression, miR19a/b overexpression by miRNA mimics also greatly inhibits the behaviors of TLR2-triggered cytokines and kinases (162). Also, miR-143 can impede the activation of TLR2, which contributes to the regulation of invasion and migration of primordial human colorectal carcinoma cells (163). By specifically targeting several proteins, including TLR4, MyD88, IRAK1, and TRAF6 (164), miR-146b will modulate the TLR4 signaling pathway. The MyD88 expression may also be controlled by miR-200a, miR-200b, and miR-200c and may alter the efficiency of the TLR4 signaling pathway, thereby affecting the inherent defenses of the host against pathogenic organisms (165). MiR-21 also inhibits MyD88 and IRAK1 expression, contributing to the upregulation during RNA virus infection of the JNK/c-Jun signaling system (166). Therefore, the synergism between miRNAs and TLRs of bovine endometrium needs further exploration during postpartum in dairy cows, particularly their function and molecular pathways in the pathogenesis of subclinical endometritis.

## Conclusion and Future Research Directions

The vast molecular analysis of bovine endometritis has a lot of reports on the utilization of LPS, a ligand of Gram-negative bacteria, (16, 17, 167–171), with a dearth of information on the use of Gram-positive bacteria ligands, such as in the case of LTA- or TAL-induced endometritis, knowing fully that most of the aberration to postpartum endometrial immune response could be because of opportunistic bacteria, majority of which are Gram-positive bacteria (19–21). Further investigation on the role of gram-positive bacteria and their ligand needs to be thorough while providing a lasting breakthrough in endometrial infection, particularly in the adoption of miRNA molecular and cellular evaluations. The new progress of immunotherapy is beneficial to prevent the indiscriminate use of antimicrobials in humans and animals through the molecular analysis of miRNA inhibitors or agonists in the drug development system to control and treat bovine endometritis. Endometrial cell miRNAs regulate inflammatory cytokine production, either contributing to mRNA degradation or inhibiting protein translation, and are involved in different biological processes and signal pathways to control gene expression (10, 48, 61, 74, 79). In this review, some miRNAs have been reported to be either upregulated or down-regulated through specific Toll-like receptors, leading to regulation of cellular signaling pathways assigned to gene modulation by degrading mRNAs in bovine endometritis (67, 68). The overexpression of some miRNAs as reported could lead to a decreased or increased level of pro-inflammatory cytokines and an increase or decrease in the expression of anti-inflammatory cytokines, suppressing or elevating the aberrant inflammatory responses that could lead to curbing or progression of bovine endometritis. The dysregulated or down-regulated expression of some miRNAs could likewise lead to an increased or decreased level of pro-inflammatory cytokines and a decreased or increased anti-inflammatory cytokine level, which could lead to progression or alleviation of endometrium inflammatory responses (172). In another vein, miRNAs implicated or incriminated in endometritis molecular pathophysiology would lead to the potential activation or deactivation of assigned Toll-like receptors through the functioning cellular signal pathways, creating medium immunopharmacological preparations or molecular modulation in the form of miRNA mimics or inhibitors that could lead to the production of an anti-inflammatory molecular therapeutic agent. The overexpression or knockdown of some sets of miRNAs symbolized their essentiality in the onset of postpartum bovine endometritis. Therefore, their molecular characterization would pave the way for their adoption in the development of miRNA utilization diagnostic protocol or potential biomarkers in early bovine endometritis detection, which may ultimately result to the molecular development of rapid test diagnostic kits.

As genetic expressive regulators, miRNAs act by inhibiting or degrading the translation of mRNAs by partially or completely combining them with the 3′-UTRs of the target mRNAs (54). They participated by acting on the posttranscriptional target gene in the inflammatory response (52, 61, 173). As reported, some genes are differentially expressed through the activities of TLRs and signaling pathways of bovine endometritis (27, 30, 59, 61, 67, 68, 100). The rapid improvement in molecular and cell spatial approaches over the course of this century has led to the discovery of several gene expressions in normal and diseased ecosystems in the endometrium. The manipulatory attenuation effect on endogenous bovine endometrium miRNA level by the administration of inhibitor or mimic substrate could change the expression of miRNA target genes and proteins. This could give a clue of the possibility for genetic selection of endometritis-resistant breed of bovine through the production of genetically edited cows with molecular and cellular resistance to bovine endometritis. Furthermore, the understanding of how exosome miRNAs influence the mechanism of bovine endometritis and somatic embryogenesis through their respective target genes has been reported to have importance on the involution of endometritis in dairy cow postpartum period and preceded a scientific finding that needs to evaluate the differentially expressed exosome microRNA, inflammatory response, and various genes involved in their cellular signal pathways, therefore the need to incorporate the sera or exosome miRNAomes in the course of abnegating bovine endometrial inflammation (174–176).

In conclusion, we suggest the need for further multi-disciplinary research work in which there is a comprehensive integration of bovine endometrial cell peculiarity in the miRNAomics during the inflammatory disease response. Differentially expressed diagnostic value must be broadly investigated to enlighten the fundamental molecular mechanisms of miRNAs involving the bovine endometrium, possible future development of new immunotherapeutic methods, and clinical trial on genes implicated in the inflammatory process through newer genetic editing techniques, such as CRIP/Cas9, which could be tested for drug and diagnostic kits development. Ultimately, a multi-disciplinary consideration of miRNA molecular pharmacological products with low toxicological or/and broad spectrum, wide safety margin, and possibly clinical trial will provide a baseline platform for the approval of the product aiming at resolving bovine endometritis.

## Author Contributions

AO and ZY contributed to the conceptualization of this work. AO, ZY, YL, BI, WS, XW, SW, and XD contributed to writing and editing. All authors have reviewed and agreed to publish this version of the manuscript.

## Conflict of Interest

The authors declare that the research was conducted in the absence of any commercial or financial relationships that could be construed as a potential conflict of interest.
